# Eighteen Years of Molecular Genotyping the Hemophilia Inversion Hotspot: From Southern Blot to Inverse Shifting-PCR

**DOI:** 10.3390/ijms12107271

**Published:** 2011-10-24

**Authors:** Liliana C. Rossetti, Claudia P. Radic, Miguel M. Abelleyro, Irene B. Larripa, Carlos D. De Brasi

**Affiliations:** Departamento de Genética, Instituto de Investigaciones Hematológicas, Academia Nacional de Medicina, Pacheco de Melo 3081, Ciudad de Buenos Aires (CP 1425), Argentina; E-Mails: pradic@hematologia.anm.edu.ar (C.P.R.); mmabelleyro@hematologia.anm.edu.ar (M.M.A.); ibl@hematologia.anm.edu.ar (I.B.L.); cdebrasi@hematologia.anm.edu.ar (C.D.D.B.)

**Keywords:** *F8*, HEMA, intron 22 inversions, IS-PCR, LD-PCR

## Abstract

The factor VIII gene (*F8*) intron 22 inversion (Inv22) is a paradigmatic duplicon-mediated rearrangement, found in about one half of patients with severe hemophilia A worldwide. The identification of this prevalent cause of hemophilia was delayed for nine years after the *F8* characterization in 1984. The aim of this review is to present the wide diversity of practical approaches that have been developed for genotyping the Inv22 (and related *int22h* rearrangements) since discovery in 1993. The sequence— Southern blot, long distance-PCR and inverse shifting-PCR—for Inv22 genotyping is an interesting example of scientific ingenuity and evolution in order to resolve challenging molecular diagnostic problems.

## 1. Introduction

Scientific development is not smoothly continuous but rather occurs in steps. There are several examples that prove the causative connection between each one of these steps and the use of novel experimental approaches. A typical example in the area of life sciences is the method of polymerase chain reaction (PCR) [1], which has revolutionized molecular diagnosis in medicine. Therefore, to tell the story of technical developments in a scientific discipline is perhaps the best way to understand it in depth. Notably, due to the molecular characteristics of the genes involved in hemophilia A and B (*i.e.*, their different molecular sizes and structure complexities) a significant number of scientists who designed and developed innovative technical approaches for mutation detection and genotyping, worked in hemophilia.

Hemophilia A (HA) (OMIM 306700) is the most severe inherited bleeding disorder that affects humans. A deficiency in FVIII clotting activity leads to this coagulopathy, which affects 1 in 5,000 males worldwide. This makes HA one of the most common X-linked inherited diseases. Virtually all patients with HA associate with deleterious mutations within the coagulation factor VIII gene (*F8*). A familial history of the disease is known in about two thirds of cases, and it appears sporadically in one third of cases. HA is expressed in a wide range of clinical severities and these differences associate with the type and location of the causative gene defect. Therefore, HA is caused by a heterogeneous spectrum of molecular defects in *F8* including deletions, large DNA inversions, nonsense mutations, ins/del-frameshifts, splice variants and a large number of missense point mutations, all of which can cause defects in the expression, secretion, and/or half-life of FVIII in circulation.

HA can be classified by the residual clotting activity of FVIII as severe, moderate or mild disease, affecting about 40%, 10% and 50% of patients with HA, respectively. As a recessive X-linked disorder, the residual activity of plasmatic FVIII in heterozygous carrier females of severe *F8* mutations is usually ~50% with respect to a non-carrier individual. Although extremely rare, homozygous females may also suffer from hemophilia in a similar way to hemizygous male patients [2]. However, most of the few cases of hemophilia expression in females are due to the coexistence of skewed Lyonization (biased Xchromosome inactivation) and the heterozygous carrier condition [3].

An international database, the HA mutation, structure, test and resource site (HAMSTeRS, URL: http://hadb.org.uk) contains extensive information, including a curated list of previously reported mutations and polymorphisms in *F8* [4]. Today, 1,209 total unique mutations of different types are collected in the worldwide database HAMSTeRS, and 797 are single-base substitutions (point mutations) (database accessed 17/10/2011). Approximately one half of the severe cases of HA are caused by inversions between a sequence located within intron 22 of the *F8* gene and sequences outside the *F8* gene.

Also characteristic of HA is the development of inhibitory antibodies against therapeutic FVIII (inhibitors) in approximately 15–35% of patients with severe HA. Particularly, FVIII inhibitors neutralize the substituted FVIII in about 21% of intron 22 inversions (a large series of patients with severe HA from the Bonn Centre, Germany) [5], a rate slightly higher than the average across all severe HA causative mutations, but lower than those cases associated with large deletions or nonsense mutations.

## 2. Milestones in Hemophilia A Mutation Characterization

### 2.1. 1984–1993: Cloning and Characterization of the Human Coagulation Factor VIII

The human *F8* gene was cloned between 1982 and 1984 [6]. At that time the gene was the largest described [6], and at approximately 187 kb, remains one of the largest (chrX:154,064,070-154,250,998, UCSC genome browser, access date 17/10/2011 [7]). Genetic mapping positioned the *F8* gene in the most distal band (Xq28) of the long arm of the X-chromosome. The *F8* gene contains 26 exons, which vary in length from 69 to 3,106 base pairs (bp). Intron sequences correspond to 177.9 kb, and are removed from the primary transcript product during splicing to generate a mature *F8* mRNA of approximately 9 kb in length that predicts a precursor protein of 2,351 amino acids. Of the larger intron sequences, we found six that are greater than 14 kb (introns 1, 6, 13, 14, 22 and 25), with intron 22 the largest at 32.8 kb in length [6].

Levinson *et al*. (1990) [8] found a curious example of a gene within a gene. Looking for transcripts within a region of Xq28 associated with several neurological disorders, the authors identified a CpG island in the largest *F8* intron. This CpG island was associated with a 1.8 kb transcript referred to as the A gene (*F8A*). The nested *F8A* gene was oriented in opposite direction to that of *F8* and contained no intervening sequences. Computer analysis of the sequence suggested that the *F8A* gene encodes a protein with the complication that codon usage analysis suggested a frameshift halfway through the gene. Freije and Schlessinger (1992) [9] subsequently demonstrated that the X-chromosome contains three copies of *F8A* and its adjacent regions, one in intron 22 and two telomeric and approximately 500 kb upstream to the *F8* gene transcription start site.

In 1992, Levinson *et al*. reported another transcript of 2.5 kb, *F8B*, that emanates from the same *F8* intron 22 CpG island as *F8A* and transcribes in the same direction as *F8*. The divergent transcripts *F8A* and *F8B* originate from within 122 bases of each start point. The newly identified 5′ exon of *F8B* in *F8* intron 22 potentially codes for eight amino acids and was spliced to *F8* exons 23–26, with the *F8* reading frame maintained [10].

Following these discoveries, Lakich *et al*. (1993) [11] pointed out that intron 22 was unusual in many respects. Containing 32.8 kb, it is the largest intron in the *F8* gene. It also contains a CpG island, located about 10 kb downstream of exon 22 [11]. This CpG island appears to serve as a bidirectional promoter for the *F8A* and *F8B* genes, which are both expressed ubiquitously in different tissues [10]. In 2001, *F8A* gene was shown to code for a 40 kD huntingtin-associated protein, termed *HAP40* [12] and is thought to be involved in the aberrant nuclear localization of the huntingtin protein in Huntington disease. The function of *F8B* is not known. Because there is no *F8B* equivalent in the mouse genome, transgenic mice that express the wild-type human *F8B* under the control of a cytomegalovirus promoter have been used to understand its function. Surprisingly, these *F8B* transgenic mice showed growth retardation, microcephaly and severe ocular defects, evidence that should encourage further studies of this protein [13].

### 2.2. 1993–2005: F8 Intron 22 Inversion Discovery and Detection

In 1993, two research groups—one led by Jane Gitschier in USA and the other one by Francesco Giannelli in UK—independently observed that one half of severe HA patients had no detectable mutation in the promoter, coding sequences or normal RNA processing signals of the *F8* gene [11,14]. Instead they revealed a unique mRNA defect that prevents the amplification of the message across the boundary between exon 22 and 23. This feature located the defect to internal regions of intron 22 and a model was proposed based on recombination between homologous *F8A* sequences located in intron 22 and upstream of the *F8* gene. Such event of homologous recombination would lead to an inversion of all intervening DNA and a disruption of the *F8* gene. Both groups presented evidence to support this model.

Unbroken *F8* gene sequences are shown as green hatched boxes and rearranged *F8* sequences as orange hatched boxes; intragenic *int22h*-1 is shown as a closed chevron; *int22h*-2 and *int22h*-3, within the arms of a large imperfect palindrome (blue), are shown as grey and open chevrons, respectively. Chimeric *int22h* sequences are denoted as [/] e.g., int22h-1/-2 represents the chimera between *h*-1 and *h*-2. Each schematic displays: (a) ISPCR based approaches developed by Rossetti *et al*. [15,16], wherein “B” represents a *Bcl* I restriction site after self-end ligation; (b) Southern blot analysis as described by Lakich *et al*. (1993) [11], wherein dashed lines show *Bcl* I restriction fragment sizes (kb); and (c) LDPCR based approaches of Bagnall *et al*. (2006) [17] (upper) and Liu *et al*. (1998) [18] (lower). Please refer to text for further explanation of details, including derivation of primers with orientation marked by arrowheads.

**Figure 1:**
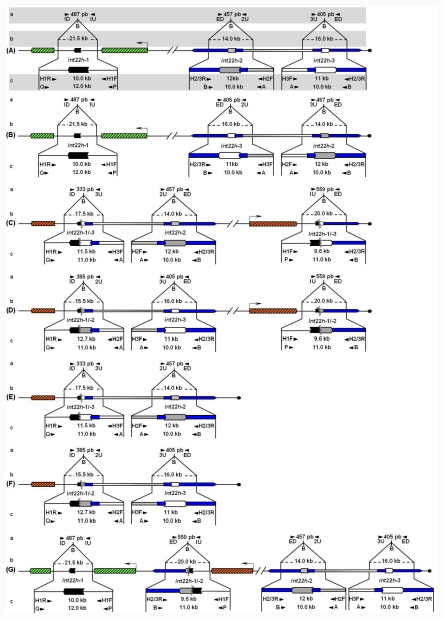
Schematic view of the *F8 int22h* normal gene regions (A, B) and *int22h*-related recombination variants (C-G). From top down, the last Mb of Xq28 is shown representing: (**A**) the normal *F8* wild-type variant *h123* (according to Xq->Xtel orientation of *int22h*-1, *h*-2 and *h*-3 sequences); (**B**) the normal *F8* wild-type variant *h132* (non-deleterious inversion polymorphism *h123*/*h132*); (**C**) HA-associated Inv22 type I originating from recombination between *h1* and *h3* on normal variant *h123* shown in (A); (**D**) HA-associated Inv22 type II originating from recombination between *h1* and *h2* on normal variant *h132* shown in (B); (**E**) HA-associated Del22 type I originating from recombination between equally oriented *int22h*-1 and *h*-3 on variant *h132* (B); (**F**) HA-associated Del22 type II originating from recombination between *int22h*-1 and *h*-2 on variant *h123* (A), (Del22 notation: NC_000023.10: g.154,118,607_154,615,713del); and (**G**) Example of non-HA-associated Dup22 originating from recombination between equally oriented *int22h*-1 and *h*-2 on variant *h123* (A), (Dup22 notation: NC_000023.10: g.154,118,607_154,615,713dup).

Lakich *et al*. (1993) further described a Southern blot assay based on *Bcl* I restriction and an *F8A* probe for which the sizes of two of the three normal hybridization bands were characteristically altered in patients presenting intron 22 inversions (Inv22) [11] [Figure 1A-D(b)]. They suggested that this assay should permit genetic prediction of HA in approximately 45% of families with severe disease [11]. Both the USA and UK groups found that this mutation occurred at the surprising rate of approximately 4 × 10^−6^ per gene, per gamete, per generation [11,14].

#### 2.2.1. First Generation: Southern Blot Analysis as the Gold Standard and Early Findings about Inv22

Southern blot analysis, as described by Lakich *et al*. (1993) [11], is still considered the reference method for Inv22 genotyping. These investigators showed that Inv22 can present two different band patterns named distal or type I, and proximal or type II (Inv22-1 and Inv22-2, respectively). Inv22 Southern blot analysis is defined by *Bcl* I enzyme restriction and a labeled probe (900 bp *Eco* RI-*Sac* I fragment of plasmid p462.6, ATCC #57203) corresponding to the *F8A* gene located within *F8* intron 22 and therefore also the two extragenic copies. Accordingly, Southern blot analysis resolves different patterns each containing three signals per allele, *i.e.*, no-Inv22 (normal allele) associated with signals of 21.5, 16, and 14 kb [Figure 1A(b) and 1B(b)]; Inv22-1, with signals of 20.0, 17.5 and 14.0 kb [Figure 1C(b)]; and Inv22-2, with signals of 20.0, 16.0, and 15.5 kb [Figure 1D(b)].

Southern blot analysis is technically robust, enables identification of all types of inversions (Inv22-1 and Inv22-2), and permits a semiquantitative evaluation of Inv22 heterozygous carrier mosaicism as in the case described by Oldenburg *et al*. (2000) [19]. However, this technique is labor-intensive requiring 8–10 days to obtain the results. Use of hazardous radiochemicals is a further disadvantage and requires authorized personnel, although use of chemiluminescence probe labeling may circumvent these potential risks.

Interestingly, Rossiter *et al*. (1994) [20] found that Inv22 originates predominantly from male germ cells and hypothesized that the presence of a second X chromosome in female meiosis would hinder the intrachromosomal non-allelic pairing required for Inv22. They presented convincing evidence supporting their findings using linkage analysis. This approach confirmed that, when occurring at the grandparents’ generation, the Inv22 was always associated with the grandfather germline (20 out of 20 informative families studied), whereas only one out of 50 mothers of sporadic cases with severe HA and the Inv22 were carriers. Contemporaneously, Tizzano *et al*. (1995) [21] observed in a Spanish population that all mothers of patients with isolated HA caused by the Inv22 resulted from carriers, also indicating that Inv22 originates in male germ cells.

Oldenburg *et al*. (2000) [19] reported the first instance of Inv22 presenting as somatic mosaicism in a female, affecting only about 50% of lymphocyte and fibroblast cells. Supposing a postzygotic *de novo* mutation as the usual cause of somatic mosaicism, the finding implies that the Inv22 mutation is not restricted to meiotic cell divisions but can also occur during mitotic cell divisions, either in germ cell precursors or in somatic cells.

Aiming to define the exact extent of the homologous sequences involved in the Inv22 crossing over event, Naylor *et al*. (1995) [22] studied an intragenic clone containing *F8* intron 22, which contains a copy of *F8A*, and two extragenic clones each with a single copy of *F8A* located by the Xq telomere using PCR amplification, chemical cleavage of mismatch (CCM) and DNA sequencing. They precisely defined the repeated region of 9.5 kb and named it *int22h*-1 (intron 22 homologous region-1) (intragenic to *F8*), and *int22h*-2 and *int22h*-3 (both extragenic to *F8*). The inversion junctions were shown to represent precise exchanges between the *int22h* repeats without insertions or deletions, thus providing conclusive evidence for homologous recombination [22]. The three copies of *int22h* were compared along more than 8 kb of their length, using CCM analysis, and found to be 99.9% similar [22]. The presence of such long and almost identical inverted repeats near the Xq telomere could account for the high frequency at which the inversions occur [22].

Antonarakis *et al*. (1995) [23] collected data on 2,093 samples from laboratories all over the world and concluded that the common inversion mutations are found in 42% of patients with severe HA (35% of Inv22 type I, 7% of type II and 0.05% of rare variants such as types IIIa and IIIb). Whereas 98% of all mothers of patients with Inv22 were carriers, data from this study was only one *de novo* inversion event occurring in maternal somatic cells for every 25 mothers of sporadic cases. When the maternal grandparental origin of inversions was examined the ratio of *de novo* occurrences in male:female germ cells was 69:1. In Argentina, De Brasi *et al*. (2000) [24] found similar figures for Inv22 type I and type II, although they did not find rare types in a group of 34 patients with severe HA (*i.e.*, 41% of total Inv22, 35% of Inv22-1 and 6% of Inv22-2). According to previous series and the evidence discussed above, the Argentine series showed that all mothers of patients with the Inv22 (and particularly those mothers of isolated cases of hemophilia) were conventional heterozygous carriers, as detected in peripheral blood DNA samples, excluding the possibility of *de novo* mutation in their gonads.

#### 2.2.2. Second Generation: Long Distance-PCR Based Approaches

During the early 1990s, the Inv22 was detectable only by labor-intensive Southern blot analysis. Therefore, a simpler, more rapid and less expensive test for Inv22 genotyping was highly desirable. Steve Sommer in USA designed a single-tube PCR assay that combines overlapping PCR [25] with long distance-PCR (LD-PCR) [26] to achieve the genetic diagnosis of Inv22 causing severe HA [18]. The new method was simple, rapid and relatively inexpensive and thus became the method of choice in many laboratories worldwide.

The inversion was detected by performing LD-PCR directly from genomic DNA with four primers that differentiate the wild-type, Inv22, and carrier genotypes. Two primers, P and Q, located within the *F8* at positions −1,212 bp and +1,334 bp flanking *int22h*-1, when combined with two different primers, A and B, flanking the two extragenic repeats *int22h*-2 and *int22h*-3 each at −167 bp and +118 bp, yield segments PQ (12 kb) and AB (10 kb) in a hemizygous individual without Inv22 and segments PB (11 kb) and AQ (11 kb) along with the 10 kb AB segment from the intact extragenic homolog in a patient with the Inv22 [Figure 1A-D(c)]. This assay does not differentiate Inv22 types I and II. Inv22 female carriers produce PQ, PB, AQ, and AB segments. In all cases, an AB segment serves as an internal control because at least one copy of *int22h*-2 or *int22h*-3 remains intact. The three long amplimers were separated by agarose gel electrophoresis 0.6% for 6–8 hours [18].

Efficient amplification of the four segments depended on three unusual modifications for LD-PCR protocols: (i) high concentrations of dimethyl-sulfoxide; (ii) addition of 7-deaza-dGTP; and (iii) high concentration of a mix of Taq and Pwo DNA polymerases [18]. However, one of the segments was amplified much more efficiently than the others under standard three-temperature cycling conditions. Consequently, to facilitate the uniform amplification of the multiple regions, subcycling-PCR was included in this protocol [27].

The accomplishment to amplify long amplimers encompassing *int22h* duplicons by Sommer’s group opened the possibility to investigate a highly informative restriction fragment length polymorphism (RFLP) of enzyme *Xba* I [28]. Notably, the contemporary reports of El-Maari *et al*. (1999) [29] and De Brasi *et al*. [30] both described methods based on hemispecific LD-PCR for *Xba* I RFLP genotyping, one primer targeting single copy DNA on *F8* intron 22 and the second primer targeting *int22h* repeat sequence. By application of the same approach of hemispecific LD-PCR for *int22h*-1 specific amplification followed by nested PCR amplification, Bowen *et al*. (2000) [31] presented a new RFLP of the restriction enzyme *Msp* I that proved heterozygous in about 46% of females of Caucasian origin. In addition, De Brasi *et al*. (2003) [32] reported streamlined genotyping of the *Xba* I and *Msp* I RFLP by use of a separate LD-PCR product obtained with primers P and Q [18] to specifically amplify *int22h*-1 followed by nested PCR. The authors reported a combined heterozygosity of 63% in Argentine population, which is an exceptionally high figure for such linked markers (750 bp).

Contrasting with the significant virtues of LD-PCR for Inv22 genotyping, amplification of such long amplimers (>10 kb) including a tract of about 3.3 kb with 79% of CG content made this assay somewhat dependent on narrow ranges of input DNA qualities, thermocycling and reagent conditions [27]. With an objective to improve Inv22 genotyping efficiency, Bowen and Kenney (2003) [33] unleashed the multiplex LD-PCR single tube reaction [18] into four separate LD-PCR reactions for each of the primer pairs PQ, PB, AQ and AB [Figure 1A-D(c)]. This separation permitted more robust amplification for each primer pair, and results were readily interpretable using standard agarose gel electrophoresis.

#### 2.2.3. Third Generation: Inverse Shifting-PCR Based Approaches

**Figure 2:**
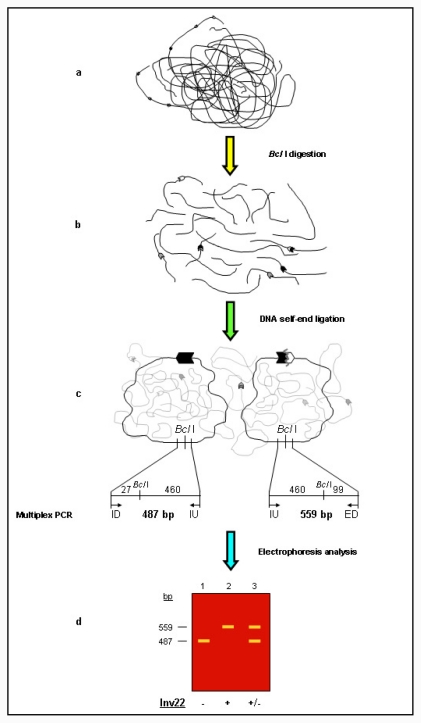
Schematic view of the inverse shifting-PCR approach. The simpler version of IS-PCR was described by Rossetti *et al*. (2005) [15] and involves three steps: (**a**) genomic DNA is subjected to restriction digestion yielding fragments (in this case *Bcl* I fragments), (**b**) restriction fragments self-end ligation (performed in large volumes), which forms (**c**) DNA circles that represent templates for a standard multiplex PCR analysis (on background some non relevant circles are shown); (**d**) PCR products from relevant circles are resolved by conventional electrophoresis. Lane 1 shows wild-type allele-specific products (-), lane 2, a male patient hemizygous for Inv22 (+), lane 3, heterozygous Inv22 carrier female (+/−). On the diagram *int22h*-1 is shown as a closed chevron; *int22h*-2 and *int22h*-3 as grey and open chevrons, respectively. Oligonucleotide primer ID indicates intragenic downstream, IU, intragenic upstream and ED, extragenic downstream.

In order to overcome the problems associated with direct amplification of *int22h* duplicons, Rossetti *et al*. (2005) [15] designed an alternative approach for Inv22 genotyping based on a variant of the classical inverse-PCR designed by Ochman *et al*. (1988) [34]. Novel Inv22 inverse-PCR analysis was inspired by the typical signal shift from 21.5 to 20 kb on Southern blot autoradiograms indicative of the presence of Inv22 type I or type II [14]. This alternative inverse-PCR based protocol included three steps: (i) *Bcl* I restriction; (ii) self-ligation of restriction fragment ends yielding *Bcl* I circles; and (iii) standard multiplex PCR analysis (Figure 2). Three years later, this approach was named inverse shifting-PCR (IS-PCR) [16] because it differs from classical inverse-PCR in that primers target at short definite distances from the site of restriction/ligation and, therefore, a sequence change associated with a particular rearrangement generates a chimeric circle that is recognized by a shift in primer usage and is ultimately reflected by the predicted size of IS-PCR product (Figure 2).

Inv22 analysis by IS-PCR was achieved using three different primers (ID, IU, ED) that yielded a 487 bp amplicon (ID/IU) for the wild-type intragenic allele and a 559 bp amplicon (ED/IU) for the Inv22 allele (Figure 2). PCR products were analyzed by standard agarose gel electrophoresis. It is important to reinforce that primers for IS-PCR were targeted to regions free of human repeats and low-complexity DNA by masking the relevant regions [15].

### 2.3. 2005–2011: Completion of the Human X-Chromosome Sequence and Definition of Hypothetical int22h-Mediated Rearrangements. Unraveling a Complex Picture

The traditional picture stated by Naylor *et al*. (1995), which reigned for a decade, proposed that both *int22h*-2 and *int22h*-3 should be in opposite orientation to *int22h*-1 on the X-chromosome [22]. In this scenario, intrachromosomal homologous recombination between *int22h*-1 and either of the two extragenic copies may result in the two varieties (types) of the recurrent inversions that cause almost half of cases of severe HA. It was believed that *int22h*-1 interacts with either the proximal (*int22h*-2), or the distal (*int22h*-3) extragenic copy, generating either Inv22 type II or type I, respectively. By this model, interaction between *int22h*-1 and *int22h*-3 would be favored over those between *int22h*-1 and *int22h*-2, thus explaining their relative frequencies (4:1, Inv22 type I: type II) (Naylor *et al*. 1995) [22].

Availability of the DNA sequence of the X-chromosome in 2005 showed that *int22h*-2 and *int22h*-3 are found within the arms of a large imperfect palindrome, and only *int22h*-3 should be involved in these inversions [35] (Figure 1A and 1B). The duplicated inverted sections (arms) are 50 kb-long and are separated by 67 kb of non-duplicated spacer sequence (Figure 1A and 1B). The *int22h*-2 and *int22h*-3 regions lie adjacent to the spacer sequence, and the more proximal of these (traditionally *int22h*-2) is in the same orientation as *int22h*-1. Therefore, recombination between *int22h*-1 and *int22h*-2 should lead to deletions or duplications rather than inversion [36].

Bagnall *et al*. (2005) suggested an attractive hypothesis to explain the relative frequencies of type I and type II inversions [36]. These investigators proposed that the large palindrome arms could recombine frequently with each other to generate an inversion of the central 67 kb segment (spacer) that expresses in the human population as a structural inversion polymorphism with frequencies of 80% and 20% for the two variants, *i.e*., *h123* and *h132*, respectively (Figure 1A and 1B).

#### 2.3.1. More on the Second and Third Generation. New Tests to Allow Comprehensive Detection of int22h-Related Rearrangements

Unfortunately, Inv22 genotyping by LD-PCR (1998) [18] and IS-PCR (2005) [15] does not permit discrimination of type I and type II Inv22 patterns (Figure 1C and 1D), nor, perhaps more importantly, do these methods allow detection of hypothesized *int22h*-related deletions (Del22) (Figure 1E and 1F) or duplications (Dup22) (Figure 1G). These limitations of rapid approaches for Inv22 genotyping opened up the possibility that molecular diagnosis may be misrepresented in some cases.

In a bid to overcome these limitations and to improve molecular diagnosis of Inv22, new protocols for Inv22 detection based on LD-PCR (2006) [37] and IS-PCR (2008) [16] were developed with the intention to identify all *int22h* rearrangements. Both of these revised protocols allow discrimination of Inv22 type I and type II patterns (Figure 1C and 1D), *int22h*-mediated deletions (Del22-1, Del22-2) (Figure 1E and 1F), and duplications (Dup22) (Figure 1G) by using complementary or additional diagnostics tests (Figure 1A-G(a) and 1A-G(c)).

Bagnall *et al*. (2006) [37] developed an LD-PCR based method for specific detection of Inv22 patterns type I and type II using a single test with four primers (named H1R, H1F, H2F and H3F) yielding a 10 kb product in normal DNA representing the intact *int22h*-1 region, and signals of 11.5 and 12.7 kb in DNA from patients with the Inv22 type I and type II, respectively [Figure 1C-D(c)]. These latter signals represent the more centromeric reciprocal of the *int22h* recombined sequences, which respectively contain part of *int22h*-3 and *int22h*-2. DNA samples from Inv22 heterozygous carriers show one of the mentioned Inv22 specific signals accompanied by the 10 kb signal seen in normal DNA that contains the non recombined copy of *int22h*-1 (Figure 1A-B(c)). As equally-oriented *int22h*-mediated duplications and/or deletions were likely to occur, it is useful to have complementary tests for detecting and distinguishing them from the inversions that cause severe HA. Consequently, Bagnall *et al*. (2006) [37] designed two complementary tests (Co1° and Co2°) using two combinations of primers. The test Co1° with primers H1F and H2/3R shows a signal of 9.6 kb from samples with the Inv22-1, Inv22-2 and Dup22 [Figure 1C(c), 1D(c) and 1G(c)], whereas Del22 type I or type II associates with an absence of signals; and Co2° with primers H1R, H3F and H2F shows an absence of signals from samples with Dup22, and a signal of 11.5 kb from either Inv22 or Del 22 type I alleles [Figure 1C(c) and 1E(c)] and 12.7 kb from either Inv22 or Del 22 type II alleles [Figure 1D(c) and 1F(c)].

Likewise, Rossetti *et al*. (2008) [16] modified their earlier reported IS-PCR protocol to resolve all *Bcl* I restriction fragments detected by classical Southern blot analysis [11]. This modified protocol enables detection of Inv22 type I and type II as well as Del22 type I and type II, and Dup22 [16]. Similar to its precursor, this modified IS-PCR protocol avoids direct amplification of *int22h* duplicons, and uses two standard PCR tests for the same substrate (*Bcl* I circles). The modified protocol includes two multiplex PCR assays: (i) a diagnostic test, which is pattern-sensitive and differentiates HA causative Inv22 and Del22 mutations from non-HA causative Dup22 and normal variants; and (ii) a complementary test intended to distinguish between Inv22 and Del22, and between Dup22 and normal allele. The diagnostic test applies primer ID with a set of three primers U (IU, 2U and 3U), enabling discrimination of normal/Dup22 allele, associated with a signal of 487 bp [Figure 1A-B(a) and 1G(a)], from Inv22/Del22 type I, with a signal of 333 bp [Figure 1C(a) and 1E(a)], and Inv22/Del22 type II with a signal of 385 bp [Figure 1D(a) and 1F(a)]. The complementary test applies primer ED with the same set of three primers U, and extends diagnostic test findings [16]. On the complementary test the normal allele shows two signals of 457 and 405 bp [Figure 1A-B(a)]; Dup22, three signals of 559, 457 and 405 bp [Figure 1G(a)]; Inv22 type I, two signals of 457 and 559 bp [Figure 1C(a)]; Del22 type I, only one signal of 457 bp [Figure 1E(a)]; Inv22 type II, two signals of 405 and 559 bp [Figure 1D(a)]; and Del22 type II shows only one signal of 405 bp [Figure 1F(a)].

IS-PCR based approaches for Inv22 genotyping have proved to enable detection and semi-quantitative assessment of carrier mosaicisms, and performed robustly over wide ranges of DNA qualities and procedural conditions, including for prenatal diagnosis [38]. The key step to achieve successful inverse-PCR protocols is the formation of DNA circles from restriction fragments by self-end ligation. Rossetti *et al*. (2008) [16] estimated the circularization efficiency range from 2–10 units of templates per circle for the formation of DNA circles of approximately 20 kb.

Despite ongoing efforts to develop technical approaches that would correctly diagnose hypothetical equally-oriented *int22h*-mediated rearrangements [36] (Figure 1), only recently have such genotypes been found. Notably, a recent paper of Abou-Elew *et al*. (2011) [39] reported three cases with signal patterns associated with Del22 (two Del22 type II and one Del22 type I) in a group of 13 Egyptian patients with HA by use of the new IS-PCR based two-test approach. This finding is somewhat unexpected taking into account that Del22 involves a loss of more than 500 kb of genomic DNA spanning a number of genes, five of which predict well characterized syndromes with specifically defined phenotypes in addition to HA [40]. In addition, Abelleyro *et al*. (2011) [40] presented a different practical approach to support the molecular diagnosis of Del22, by stating the absence or presence of a number of evenly spaced STS (sequence tagged sites) in order to confirm or to exclude the Del22 associated gap, respectively.

## 3. Significance of the Human Genome Project for Inv22 Detection and Diagnosis of HA

This review of an important area of molecular diagnosis in humans clearly shows that the development of new genotyping methods for *int22h*-mediated rearrangements relies on the extraordinary achievements of the Human Genome Project. In particular, the completion and release of the human X-chromosome sequence [35] permitted an accurate definition of both hypothetical and well established *int22h*-mediated rearrangements. De Brasi and Bowen (2008) [41] made use of widely available bioinformatic resources, such as BLAST (basic local alignment search tool) [42] and Smith-Waterman [43] algorithms, to calculate the exact extent of *int22h* duplicons and their nucleotide sequence differences, the size and location of the large inverted repeats as the arms of the large imperfect palindrome *int22h*-2 and *int22h*-3, and to precisely define the full range of *int22h*-mediated rearrangements (*i.e.*, Inv22, Del22 and Dup22).

Moreover, it has been demonstrated that bioinformatic resources developed from Human Genome Project initiatives, are providing essential tools for the accurate design of experiments in the molecular biology field. The recent design of both LD-PCR and IS-PCR based approaches for genotyping *int22h*-mediated rearrangements offers two clear examples of this [37,16]. IS-PCR-based genotyping for *int22h*-related rearrangements was designed using the latest version at that time of the nucleotide sequence of the human X chromosome, GenBank accession NC_000023.9, nt.153,500,000-154,387,415, which encompassed the entire *F8*, and the centromeric and telomeric arms of the 168 kb imperfect palindrome in which *int22h*-2 and *int22h*-3 are located.

Sequence analysis required extensive application of *in silico* tools including *Bcl* I restriction mapping, DNA sequence alignments, repeat masking, virtual circle formation by self-end ligation and oligonucleotide primer selection. Additionally, in order to illustrate the usefulness of bioinformatic developments in the area of molecular diagnostic medicine even further, the repeat masker web server (URL: http://www.repeatmasker.org) was used to map human repeats throughout all relevant sequences in order to limit the primer target sites to human repeat-free regions [15,16].

In summary, three genotyping methods presently allow Inv22 analysis to discriminate all *int22h*-mediated rearrangements (*i.e.*, Inv22, Del22 and Dup22), thus reducing potential diagnostic mistakes to a minimum. These methods include Southern blot analysis by Lakich *et al*. (1993) [11], the discriminative three-test based LD-PCR by Bagnall *et al*. (2006) [37], and the discriminative two-test based IS-PCR by Rossetti *et al*. (2008) [16]. These three generations of molecular methods for genotyping the hemophilia inversion hotspot are valuable examples of international cooperation, experimental ingenuity and, ultimately, scientific evolution to solve challenging molecular diagnostic problems.
